# Supplementary material for: Predicting changes in language skills between 2 and 3 years in the EDEN mother–child cohort
Source: PeerJ. 2014 Apr 1;2:e335. doi: 10.7717/peerj.335 (PMC3976115; doi:10.7717/peerj.335)
Supplement: Table S1 [file peerj-02-335-s001.docx]

**Supplementary table**: Attrition analysis of children without exclusion criteria (N = 1886) [mean (SD) or %].

|  |  | Sample  with LC-3 available  [Group 1] N = 1031 | Children excluded  because LC-3 was missing [Group 2] N = 370 | Children excluded  because CDI-2 was missing [Group 3] N = 485 | Wald F Test Group 1  vs. Group 2 *(p value)* | Wald F Test Group 1 + Group 2  vs. Group 3 *(p value)* |
| --- | --- | --- | --- | --- | --- | --- |
| **Child** | |  |  |  |  |  |
|  | Male gender, % | 52 | 51 | 48 | 0.1 (0.8) | 0.4 (0.5) |
|  | Birth weight, *kg* | 3.30 (0.49) | 3.23 (0.55) | 3.26 (0.53) | 5.2 (0.02) | 1.1 (0.3) |
|  | Birth term, *weeks* | 39.30 (1.65) | 39.09 (1.80) | 39.15 (1.69) | 4.4 (0.04) | 0.9 (0.3) |
| **Mother** | |  |  |  |  |  |
|  | Maternal age at birth of child, *years* | 29.52 (4.67) | 28.78 (4.90) | 28.15 (5.17) | 6.6 (0.01) | 20.6 (<0.001) |
|  | Alcohol during pregnancy (>3 units/week), % | 8 | 7 | 6 | 0.6 (0.4) | 0.6 (0.4) |
|  | Tobacco during pregnancy, % | 21 | 27 | 38 | 5.6 (0.02) | 42.1 (<0.001) |
| **Family history of language delay**, % | | 12 | 16 | 15 | 16.0 (<0.001) | 0.2 (0.7) |
| **Breastfeeding** | |  |  |  |  |  |
|  | Initiation, % | 73 | 72 | 72 | 0.0 (0.9) | 0.1 (0.7) |
|  | Duration, *months* | 4.71 (3.75) | 4.19 (3.58) | 3.92 (3.12) | 3.9 (0.05) | 8.6 (0.003) |
| **Child's environment** | |  |  |  |  |  |
|  | Household income (euros), % |  |  |  | 7.48 (0.02) | 38.0 (<0.001) |
|  | <2300 | 40 | 47 | 59 |  |  |
|  | 2300-3000 | 30 | 23 | 21 |  |  |
|  | >3000 | 30 | 30 | 20 |  |  |
|  | Parental education, *years* | 13.67 (2.29) | 13.25 (2.42) | 12.58 (2.33) | 9.1 (0.003) | 60.4 (<0.001) |
|  | Caretaker, % |  |  |  | 36.4 (<0.001) | 276 (<0.001) |
|  | Nursery | 22 | 22 | 1 |  |  |
|  | Other | 47 | 31 | 2 |  |  |
|  | Family | 9 | 14 | 1 |  |  |
|  | Mother | 21 | 33 | 97 |  |  |
|  | Number of older siblings, % |  |  |  | 2.0 (0.4) | 20.0 (<0.001) |
|  | 0 | 48 | 44 | 36 |  |  |
|  | 1 | 35 | 38 | 40 |  |  |
|  | >1 | 17 | 17 | 24 |  |  |
|  | Bilingualism, % | 10 | 9 | 0 | 0.2 (0.6) | 20.1 (0.001) |
|  | Frequency of maternal stimulation^#^ |  |  |  |  |  |
|  | between 0-2 years | 3.32 (0.72) | 3.34 (0.71) | 2.07 (0.34) | 0.2 (0.6) | 318.9 (<0.001) |
|  | between 2-3 years | 3.19 (0.70) | 3.20 (0.48) | 3.18 (0.37) | 0.0 (0.9) | 0.1 (0.8) |
|  | between 0-3 years | 3.25 (0.61) | 3.27 (0.50) | 2.78 (0.36) | 0.2 (0.7) | 212.8 (<0.001) |
|  | Pre-elementary schooled |  |  |  |  |  |
|  | Yes, % | 67 | 51 | 67 | 16.0 (<0.001) | 0.2 (0.7) |
|  | School attendance, *months* | 3.07 (3.34) | 1.49 (2.59) | 2.77 (3.39) | 11.4 (<0.001) | 0.6 (0.5) |
| **Recruitement centre** (Poitiers), % | | 52 | 36 | 47 | 25.8 (<0.001) | 3.4 (0.06) |
| **Language measures** | |  |  |  |  |  |
|  | CDI-2 | 61.12 (28.72) | 54.80 (30.19) | na | 12.6 (0.004) | na |
|  | LC-3 | 0.01 (1.01) | na | na | na | na |
| ^#^ On a scale of 1 (shared activities less than once per week) to 5 (shared activities nearly every day). The frequency of maternal stimulation between 0 and 3 years correspond to the average of this measure between 0 and 2 years and between 2 and 3 years. Abbreviations: CDI-2: MacArthur Communicative Development Inventory at 2 years. LC-3: Language component at 3 years. SD: Standard deviation. na: non applicable. | | | | | | |
